# Analysis of the work, health and well-being experience of immigrant personal support workers in minority language contexts in Canada: Scoping review protocol

**DOI:** 10.1371/journal.pone.0354960

**Published:** 2026-07-31

**Authors:** Léonel Philibert, James Plaisimond, Judith Lapierre, Soutongnoma Safiata Kaboré, André Nguwo Osomba, Luula Mariano, Frédéric Bergeron, Gbetogo Maxime Kiki, Gisèle Mandiangu Ntanda

**Affiliations:** 1 Université de l’Ontario Français, Toronto, Ontario, Canada; 2 Université Laval, Faculté des sciences infirmières, Québec, Québec, Canada; 3 Institut du savoir Montfort, Ottawa, Ontario, Canada; 4 Centre de recherche du CHU de Québec-Université Laval, Québec, Québec, Canada; 5 VITAM: Centre de recherche en santé durable, Québec, Canada; 6 Université de Montréal, Québec, Canada; 7 FRIE (Formation-Recherche-Innovation et Évaluation Inc.), Toronto, Ontario, Canada; 8 Bibliothèque-Direction des Services-Conseil, Université Laval, Québec, Québec, Canada; 9 Centre Interdisciplinaire de Recherche en Réadaptation et Intégration Sociale (Cirris), Québec, Québec, Canada; 10 Université du Québec en Outaouais, Département des sciences infirmières, Saint-Jérôme, Québec, Canada; University of Saskatchewan, CANADA

## Abstract

**Background:**

The aging Canadian population has led to an increase in Canada’s use of home and community care services. In this context, the healthcare system relies heavily on personal support workers who work in various healthcare settings. A significant proportion of these workers are immigrants, and many live in linguistic minorities. Despite their pivotal role, the experiences and working conditions of these personal support workers are underrepresented in the scientific literature, specifically in intersectional analyses. This scoping review aims to examine the work experiences, health conditions, and well-being of immigrant personal support workers in Canada who work in a minority language setting.

**Eligibility criteria:**

Studies addressing the work experiences, health conditions, and well-being of immigrant personal support workers in Canada who work in a minority language setting, and those published in English or French. There will have no restrictions on publication date.

**Method:**

This review will follow the JBI recommendations for scoping reviews and incorporate the PRISMA-ScR checklist. We will develop an adapted search strategy for five relevant databases (Medline [Ovide], Web of Science, Embase, CINAHL, and Google Scholar). Two independent reviewers will select full-text articles based on pre-established inclusion criteria and extracted relevant data. The results will be presented in accordance with the PRISMA-ScR (Preferred Reporting Items for Systematic Reviews and Meta-analyses Extension for Scoping Reviews) guidelines. This scoping review contributes to expanding knowledge about the professional, health, and social realities of immigrant PSWs in Canada’s linguistic minority communities.

**Review registration:** OSF https://osf.io/wdx63

## Introduction

The rapid aging of Canada’s population represents a major challenge for the healthcare system, as it increases the demand for health care and social services. For the first time, the proportion of people aged 65 and over exceeded that of young people under 15 years old [1]. This demographic transition is accompanied by an increase in the need for care of people living with chronic diseases and complex comorbidities [2]. This is in addition to the support needs of people with disabilities, who may require partial or complete assistance. To address this growing need while controlling costs, Canada tends to shift its care options away from hospitals towards non-hospital settings, such as long-term care facilities, retirement homes, and home care [3].

In these settings, most primary care is provided by personal support workers (PSWs), also known as patient support workers [2,4–6]. They perform essential tasks such as helping with hygiene, feeding, mobility, comfort, and psychosocial support for users [7]. Indeed, they work primarily with the elderly or people with disabilities, many of whom also have severe cognitive impairments [7,8]. As PSWs provide their services to a fairly vulnerable clientele making their work particularly challenging on the physical, emotional, and relationally [9].

Moreover, the health and social services sectors are among Canada’s largest employers for immigrant workers [10,11]. In this context, despite playing a central role in the delivery of care in non-hospital settings, PSWs, who are mostly women with an immigrant background and who come from socioeconomically precarious contexts, are considered a marginalized group within the professional hierarchy [7,12]. This corresponds to studies that show that migratory status, whether temporary or permanent, can affect access to social rights, job stability, and health services [11,13]. PSWs with temporary immigration status are often vulnerable to exploitation, and some have employer-specific work permits [11,13]. Asking for better working conditions can result in the revocation of their permit and immediate deportation to their home country. In addition, immigrant PSWs operate in environments characterized by dynamic linguistic shifts and complex cultural contexts that can influence their professional experiences, mental and physical health, and overall well-being. In Canada, which has an expanding ethnolinguistic diversity, these workers may find themselves in a linguistic or cultural minority position, even if their specific circumstances are not officially recognized by public policies [14]. According to previous studies [13,15], this framework’s minority position will likely generate additional challenges related to communication, professional integration, recognition of skills, and access to appropriate health services. Other studies have highlighted that the precarious working conditions, emotional overload, and systemic discrimination that they routinely experience are likely to affect their well-being and quality of life [15,16].

The COVID-19 pandemic has highlighted the essential role of PSWs as well as their deep vulnerabilities, particularly due to their precarious migration status and prolonged exposure to health risks [17,18]. For example, the infection rate with SARS-CoV-2 was two or three times higher among PSWs than that of nurses and doctors [19]. The inequalities experienced by immigrant PSWs are often the result of linguistic, cultural, and structural factors [20,21], with language surfacing as a key determinant of PSWs’ access to care, quality of professional interactions, and sense of belonging [2].

Despite their overrepresentation in this field, immigrant PSWs remain underrepresented in scientific literature. To our knowledge, existing studies only partially explore the impact of linguistic and cultural minority status on health, well-being, and career trajectory [13,18,21].

To address this gap, this research project will aim to examine the work, health, and well-being experiences of immigrant PSWs operating as linguistic and cultural minorities in Canada. This will help us better understand the interactions between migration, language, working conditions, and health from an intersectional perspective.

### Review question

This scoping review following questions are:

1. What are the work experiences of immigrant PSWs working in minority language and cultural contexts in Canada?
2. How do working conditions influence the health and well-being of PSWs with an immigrant background, operating in linguistic and cultural minority contexts in Canada?

### Inclusion criteria

The inclusion criteria will be in line with the JBI Framework, including population, concept and context (PCC) [22]. Articles addressing the work experiences, health conditions, and well-being of immigrant personal support workers in Canada working in minority language settings will meet these criteria. The selection criteria are summarized in Table 1.

**Table 1:** PCC Model. The different keywords used for each inclusion criterion.

Component	Definition
Population (P)	Persons with a migrant background, regardless of age, gender, ethnicity, language, or immigration status (permanent residents, refugees, asylum seekers, temporary workers, students, etc.).
Concept (C)	Professional experience (career, recognition, integration), working conditions (remuneration, stability, rights, workload), state of health (physical, mental, access to care) and well-being at work (pleasure, self-relation, autonomy and room for manoeuvre within the organization one works with).
Background (C)	Canadian context, including linguistic minority situations and multicultural environments in provinces with an English-speaking majority or a French-speaking majority.

#### Types of sources.

This review will examine research studies that explore the work experiences, health, and well-being of immigrant personal support workers in Canada, who primarily speak minority languages. The studies can be qualitative, quantitative, or mixed methods and must be published in English or French, with no restriction on the publication date. To identify additional references, we will consult systematic and scoping reviews relevant to the topic. However, these references will not include in the data-extraction process.

### Methods

We will conduct a scoping review following the JBI recommendations for scoping review(22). The goal of a scoping review is to explore a research question by mapping the key concepts, types of available scientific evidence, and gaps in the area being studied [23]. According to the methodological framework proposed by the JBI, this process takes place in four (4) steps: 1) defining the research questions, 2) identifying and selecting relevant studies, 3) extracting and organizing data, and 4) analyzing, synthesizing, and mapping the results. This review also relies on the PRISMA-ScR checklist (S1 file) to ensure transparency and rigor in the review process [24].

#### Search strategy.

The search strategy (Appendix 1) was developed in collaboration with a librarian at Université Laval (FB) and validated by research team members in October 2025. The four main databases targeted were MEDLINE (Ovide), Embase, CINAHL, and Web of Science (see Appendix I). Furthermore, additional searches were conducted on Google Scholar to increase the chances of finding other publications, particularly those in French. As French is one of Canada’s two official languages, there is a growing number of French-language journals that are not indexed in the four main databases. Google Scholar makes it possible to locate the publications of these journals dealing with the question under study.

We will also consult gray literature such as government reports, institutional websites, theses, conference abstracts, and scientific publications. A manual search will be conducted, and the reference lists of relevant studies will be searched for additional references that could expand the corpus.

This strategy will be adjusted iteratively throughout the process based on the results achieved and the adjustments required to ensure sensitivity and relevance. To maximize the completeness of the search, broad and inclusive keywords will be favored over overly specific terms. Indeed, using a combination of free terms and controlled vocabulary will optimize searches in various databases while minimizing the risk of omission [25]. Finally, the search strategy will target articles published in French or English without any restrictions on the date. The article selection, the quality evaluation of the selected articles, the data extraction, and the drafting of the scoping review will take place from January to July 2026.

#### Study selection.

All results from the database searches will be imported into Covidence (Veritas Health Innovation, Melbourne, Australia), an online collaboration platform dedicated to the management of systematic reviews. This platform automatically eliminates duplicates before the selection stage begins.

The selection of studies will be conducted in two stages. First, two evaluators will conduct a preliminary assessment of the title and abstract of each article in the database, based on the criteria established for inclusion in the study in order to select eligible studies. Then, after the preliminary screening, the reviewers will read the full text to select eligible studies according to the established criteria. Two independent evaluators will perform this selection in a parallel and rigorous manner. Any disagreement will be resolved by consensus, or in the event of a persistent divergence, a third researcher from the team will be invited to decide on the divergence.

The full process for identifying and selecting studies will be presented using a PRISMA scoping review Flow Chart [26], illustrating the number of records identified, retained, and excluded as well as the reasons for exclusion at the full-text stage.

#### Data extraction.

Data extraction and analysis will begin in March 2026. We will develop a standardized extraction grid (S2 File) to collect data, including the following:

- Descriptive data (title, year, authors, country);
- Study design and methodology;
- Context (location, health care or community context);
- Population (sample size, characteristics);
- Type of results (qualitative, quantitative or mixed);
- Workers’ health and well-being
- Gender/sex;
- Migration status (permanent resident, refugee claimant, closed work permit, open work permit, study permit);
- Work experiences and working conditions of immigrant PSWs in Canada.

The data extraction grid will be adapted and revised as necessary throughout the process to ensure the relevance and consistency of the collected information. The final version of this grid will be presented in the S2 File).

Members of the research team will carry out the extraction process, while others will verify it to ensure that the extracted content is rigorous and valid. The data collected will focus on the characteristics of the studies (population, context, objectives, main results, etc.) and on the relevant elements for the studied issues.

In keeping with the exploratory nature of a scoping review, the objective of which is to examine the breadth, diversity, and gaps in the existing literature on a given theme [27,28], so we will not assess the quality of the studies. However, we will document elements concerning the methodology of the included studies using the mixed methods appraisal tool (MMAT) version 2018 which allows to simultaneously evaluate qualitative, quantitative and mixed studies [29]. It is an excellent tool for evaluating and critiquing primary education.

#### Data analysis and presentation of results.

We will conduct descriptive analyses to systematically organize and visualize key findings from the selected documents. First, in keeping with the research questions of this study, we will categorize the results by theme, including work experiences, working conditions, reported health status, and well-being of immigrant PSWs in Canada in a narrative synthesis. Next, we will do an intersectional analysis to examine how language, working conditions, race, workplace, migration status, and others intersect to influence the health and well-being of PSWs. Finally, we will create graphical representations to illustrate the main ideas according to the nature of the data analyzed.

## Results

The results of this scoping review will be presented in accordance with the PRISMA-ScR (Preferred Reporting Items for Systematic Reviews and Meta-Analysis Extension for Scoping Reviews) guidelines to ensure transparency, traceability, and methodological rigor of the synthesis process [26]. The results of this study will inform policy, workforce planning, or interventions to support immigrant PSW working in minority language contexts in Canada and around the world. The scoping review for the publication is scheduled to be submitted in August 2026.

## Discussion

This review will provide a better understanding of the complex dynamics that shape the experience of PSWs with immigrant backgrounds in Canada. By shedding light on the specific realities of these workers, this synthesis will help fill a blind spot in the scientific literature that remains little explored. This study will shed light on the impacts of PSWs’ working conditions on their health and well-being, and will also propose recommendations for improving the practice experience of these health care workers, known as the guardian angel, during the COVID-19 pandemic in Canada.

## Conclusion

This study will help identify existing research gaps on PSWs in Canada. This scoping review will contribute to enriching knowledge on the professional, health, and social realities of immigrant PSWs in Canada. By highlighting the issues faced by this specific population, the development of more equitable, inclusive, and culturally sensitive workplaces will be promoted. The results obtained will help guide public policies and interventions towards better recognition and appropriate support for these essential health system workers.

## Supporting information

S1 File Checklist. Preferred reporting items for systematic reviews and meta-analyses extension for Scoping Reviews (PRISMA-ScR) checklist.
(DOCX)

S2 File Appendix I Databases search strategy: The search equations carried out in the different databases and their results. (DOCX)
