# Supplementary material for: Analysis of the work, health and well-being experience of immigrant personal support workers in minority language contexts in Canada: Scoping review protocol
Source: PLoS One. 2026 Jul 31;21(7):e0354960. doi: 10.1371/journal.pone.0354960 (PMC13426990; doi:10.1371/journal.pone.0354960)
Supplement: S2 File — (DOCX) [file pone.0354960.s002.docx]

**Appendix I—Databases search strategy: The search equations carried out in the different databases and their results.**

**Medline (OVID)**

**Date of the search:** 10-10-2025

**Database limit:** no database limit has been applied.

| **#** | **Search terms** | **Results** |
| --- | --- | --- |
| 1 | Allied Health Personnel/OR Home Health Aides/OR Nursing Assistants/  OR Caregivers/OR ([nurs* OR care OR Healthcare OR Home] adj 1 [aid OR aide? OR assistant? OR Auxiliar*]).ti,ab,kf OR (support adj 1 [Worker? OR Professional OR Personnel]).ti,ab,kf OR caregiver?.ti,ab,kf | 158,013 |
| 2 | exp Emigrants and Immigrants/OR Emigrant*.ti,ab,kf OR Immigrant*.ti,ab,kf  OR Foreign*.ti,ab,kf OR Migrant?.ti,ab,kf OR newcomer?.ti,ab,kf OR Refugee?.ti,ab,kf OR Asylum?.ti,ab,kf OR (residenc* adj1 (permit OR permanent OR temporary)).ti,ab,kf | 182,552 |
| 3 | exp Canada/OR Canada.ti,ab,kw,kf OR canadian.ti,ab,kw,kf OR Alberta.ti,ab,kw,kf OR “British Columbia”. ti,ab,kw,kf OR Manitoba.ti,ab,kw,kf OR New Brunswick.ti,ab,kw,kf OR Newfoundland.ti,ab,kw,kf OR “New Foundland*”. ti,ab,kw,kf OR “Northwest Territor*”. ti,ab,kw,kf OR “Nova Scotia”. ti,ab,kw,kf OR Nunavut.ti,ab,kw,kf OR Ontario.ti,ab,kw,kf OR “Prince Edward Island”. ti,ab,kw,kf OR Quebec.ti,ab,kw,kf OR Saskatchewan.ti,ab,kw,kf OR Yukon.ti,ab,kw,kf OR Toronto.ti,ab,kw,kf OR Montreal.ti,ab,kw,kf OR Calgary.ti,ab,kw,kf OR Ottawa.ti,ab,kw,kf OR Edmonton.ti,ab,kw,kf OR Winnipeg.ti,ab,kw,kf OR Fredericton.ti,ab,kw,kf OR “St John*”. ti,ab,kw,kf OR Vancouver.ti,ab,kw,kf OR Halifax.ti,ab,kw,kf OR Victoria.ti,ab,kw,kf OR Charlottetown.ti,ab,kw,kf OR Regina.ti,ab,kw,kf OR Saskatoon.ti,ab,kw,kf OR Whitehorse.ti,ab,kw,kf OR Iqaluit.ti,ab,kw,kf OR Yellowknife.ti,ab,kw,kf | 350,636 |
| 4 | 1 AND 2 AND 3 | 123 |

**Embase (Embase.com)**

**Date of the search:** 10-10-2025

**Database limit:** no database limit has been applied.

| **#** | **Search terms** | **Results** |
| --- | --- | --- |
| 1 | “nursing assistant”/de OR 'health auxiliary'/de OR 'caregiver'/de OR 'formal caregiver'/de OR ([nurs* OR care OR Healthcare OR Home] NEAR/1 [aid OR aide$ OR assistant$ OR Auxiliar*]):ti,ab,kw OR (support NEAR/1 [Worker$ OR Professional OR Personnel]):ti,ab,kw OR caregiver$:ti,ab,kw | 236,693 |
| 2 | “migrant”/exp OR “foreigner”/exp OR “refugee”/exp  OR Emigrant*:ti,ab,kw OR Immigrant*:ti,ab,kw OR Foreign*:ti,ab,kw OR Migrant$:ti,ab,kw OR newcomer$:ti,ab,kw OR Refugee$:ti,ab,kw OR Asylum$:ti,ab,kw OR (residenc* NEAR/2 [permit OR permanent OR temporary]):ti,ab,kw | 228,028 |
| 3 | “Canada”/exp OR Canada:ti,ab,kw OR canadian:ti,ab,kw OR Alberta:ti,ab,kw OR “British Columbia”:ti,ab,kw OR Manitoba:ti,ab,kw OR New Brunswick:ti,ab,kw OR Newfoundland:ti,ab,kw OR “New Foundland*”:ti,ab,kw OR “Northwest Territor*”:ti,ab,kw OR “Nova Scotia”:ti,ab,kw OR Nunavut:ti,ab,kw OR Ontario:ti,ab,kw OR “Prince Edward Island”:ti,ab,kw OR Quebec:ti,ab,kw OR Saskatchewan:ti,ab,kw OR Yukon:ti,ab,kw OR Toronto:ti,ab,kw OR Montreal:ti,ab,kw OR Calgary:ti,ab,kw OR Ottawa:ti,ab,kw OR Edmonton:ti,ab,kw OR Winnipeg:ti,ab,kw OR Fredericton:ti,ab,kw OR “St John*”:ti,ab,kw OR Vancouver:ti,ab,kw OR Halifax:ti,ab,kw OR Victoria:ti,ab,kw OR Charlottetown:ti,ab,kw OR Regina:ti,ab,kw OR Saskatoon:ti,ab,kw OR Whitehorse:ti,ab,kw OR Iqaluit:ti,ab,kw OR Yellowknife:ti,ab,kw | 211,485 |
| 4 | #1 AND #2 AND #3 | 78 |

**CINAHL**

**Date of the search:** 10-10-2025

**Database limit:** no database limit has been applied.

| **#** | **Search terms** | **Results** |
| --- | --- | --- |
| 1 | MH “Allied Health Personnel” OR MH “Certified Nursing Assistants” OR MH “Home Health Aides” OR MH “Health Personnel, Unlicensed” OR MH “Caregivers” OR XB ([nurs* OR care OR Healthcare OR Home] N1 [aid OR aide# OR assistant# OR Auxiliar*]) OR XB (support N1 [Worker# OR Professional OR Personnel]) OR XB caregiver# | 123,260 |
| 2 | MH “Emigration and Immigration” OR MH “Immigrants+” OR MH “Migrants” OR MH “Refugees” OR XB Emigrant* OR XB Immigrant* OR XB Foreign*  OR XB Migrant# OR XB newcomer# OR XB Refugee# OR XB Asylum# OR XB (residenc* N1 (permit OR permanent OR temporary)) | 63,922 |
| 3 | MH “Canada+” OR TI Canada OR AB Canada OR AF Canada OR TI canadian OR AB Canadian OR TI Alberta OR AB Alberta OR TI “British Columbia” OR AB “British Columbia” OR TI Manitoba OR AB Manitoba OR TI “New Brunswick” OR AB “New Brunswick” OR TI Newfoundland OR AB Newfoundland OR TI “New Foundland*” OR AB “New Foundland*” OR TI “Northwest Territor*” OR AB “Northwest Territor*” OR TI “Nova Scotia” OR AB “Nova Scotia” OR TI Nunavut OR AB Nunavut OR TI Ontario OR AB Ontario OR TI “Prince Edward Island” OR AB “Prince Edward Island” OR TI Quebec OR AB Quebec OR TI Saskatchewan OR AB Saskatchewan OR TI Yukon OR AB Yukon OR TI Toronto OR AB Toronto OR TI Montreal OR AB Montreal OR TI Calgary OR AB Calgary OR TI Ottawa OR AB Ottawa OR TI Edmonton OR AB Edmonton OR TI Winnipeg OR AB Winnipeg OR TI Fredericton OR AB Fredericton OR TI “St John*” OR AB “St John*” OR TI Vancouver OR AB Vancouver OR TI Halifax OR AB Halifax OR TI Victoria OR AB Victoria OR TI Charlottetown OR AB Charlottetown OR TI Regina OR AB Regina OR TI Saskatoon OR AB Saskatoon OR TI Whitehorse OR AB Whitehorse OR TI Iqaluit OR AB Iqaluit OR TI Yellowknife OR AB Yellowknife | 397,287 |
| 4 | S1 AND S2 AND S3 | 153 |

**Web of Science**

**Date of the search:** 10-10-2025

**Database limit:** no database limit has been applied.

| **#** | **Search terms** | **Results** |
| --- | --- | --- |
| 1 | TS=([nurs* OR care OR Healthcare OR Home] NEAR/1 [aid OR aide$ OR assistant$ OR Auxiliar*]) OR TS=(support NEAR/1 [Worker$ OR Professional OR Personnel]) OR TS=caregiver$ | 181,297 |
| 2 | TS=Emigrant* OR TS=Immigrant* OR TS=Foreign* OR TS=Migrant$ OR TS=newcomer$ OR TS=Refugee$ OR TS=Asylum$ OR TS=(residenc* NEAR/2 [permit OR permanent OR temporary]) | 640,906 |
| 3 | TS=(Canada OR canadian OR Alberta OR “British Columbia” OR Manitoba OR New Brunswick OR Newfoundland OR “New Foundland*” OR “Northwest Territor*” OR “Nova Scotia” OR Nunavut OR Ontario OR “Prince Edward Island” OR Quebec OR Saskatchewan OR Yukon OR Toronto OR Montreal OR Calgary OR Ottawa OR Edmonton OR Winnipeg OR Fredericton OR “St John*” OR Vancouver OR Halifax OR Victoria OR Charlottetown OR Regina OR Saskatoon OR Whitehorse OR Iqaluit OR Yellowknife) | 712,579 |
| 4 | #1 AND #2 AND #3 | 260 |

**Google Scholar (https://harzing.com/resources/publish-or-perish)**

**Date of the search:** 10-10-2025

**Database limit:** Only up to the 30 first results have been considered; citations and patent options have been removed.

| **#** | **Search** | **# Results screened** |
| --- | --- | --- |
| 1 | Canada Préposé\|préposée\|préposés bénéficiaires immigrant\|immigrante\|immigrants | 30 |
| 2 | Canada Préposé\|préposée\|préposés bénéficiaires migrant\|migrants\|migratoire | 30 |
| 3 | Canada "aide soignant"\|"aide soignante" immigrant\|immigrante\|immigrants | 30 |
|  | **Total number of results** | **90** |
